# Spinal trauma: A challenge ahead

**DOI:** 10.4103/0019-5413.36984

**Published:** 2007

**Authors:** Anil K Jain

**Affiliations:** Editor- Indian Journal of Orthopaedics, Professor of Orthopaedics, University College of Medical Sciences, University of Delhi, Delhi - 110 095, India. E-mail: dranilkjain@gmail.com

The annual incidence of traumatic spinal cord injury (SCI) in developed countries varies between 11.5-53.4 per million population.^1^ Cervical spine trauma accounts for 55% of all spinal cord injuries.^2^ Injury to the vertebral column with or without SCI commonly occurs in a polytraumatized patient. Road traffic accident is the most common mode of trauma. Low-energy household injuries are one of the most common modes in developing countries. Most spinal trauma occurs in young patients in the productive age group.^3^ Spinal trauma is more than a vertebral injury. Spinal cord injury in association with trauma to the cervical spine can compromise respiratory and cardiovascular function as a result of neurological deficit. The injury to the vertebral column not only disrupts the load-bearing column and stability of the torso but also injures neural elements which are housed in the spinal canal. The resultant paraplegia or quadriplegia do not have as good a prognosis for neural recovery as observed in paraplegia/ quadriplegia secondary to disease of vertebral column or neural tissues.

The spinal cord and cauda equina are injured by disruption of neural tissues due to violation of the spinal canal by bony missiles (pieces) entering into the spinal canal as a result of impact. The acute compression of the spinal cord produces histologically appreciable and irreversible secondary changes in the spinal cord. The disrupted vertebral column is potentially unstable and hence neural tissues are at risk of insult during the post injury period. The neural tissues may get injured inadvertently when the patient is transported or treated until the vertebral column is restored and stabilized surgically or by healing. The neural tissues are at risk of late insult in a potentially unstable spine which has not been stabilized surgically or spontaneously.

More than 50% of spinal trauma is preventable. The most common cause is fall from a tree or a roof of the building, due to a dive in a shallow pond in villages or while working as a laborer on a high-rise building without safety precautions. Most of the houses have no parapets. The persons sleeping on the rooftops fall while sleeping. These are all preventable injuries and can be avoided if people are educated and safety norms are practiced mandatorily. Unfortunately, we do not have population-based data for such preventable injuries to stress on the law-enforcing agencies to ensure implementation of stringent safety norms.

Once an injury occurs in a rural setting there are no mechanisms for safe evacuation of these patients. The patients are transported by various modes of transport on bumpy and kuccha roads by relatives. They have to cover 100 km or more to reach a district hospital. Irreparable damage is caused to the spinal cord or cauda equina in this manner even if it had escaped damage during trauma. Most of the district hospitals have no facilities to treat spinal injury patients. Some of these patients even remain untreated. The patients with a good financial background reach a hospital where facilities to treat such injuries are available, with a delay of five to seven days. We get most of our patients with deformed spine with concomitant complications of SCI such as pressure sores, urinary retention and infection, deep vein thrombosis. The physical, personal, financial and social impact of spinal cord injury is such that most patients are lost in followup or succumb to life-threatening complications of SCI.

Ours is a country of paradoxes. On the one hand we have private hospitals with state-of-the-art treatment facilities provided at an exorbitant cost, not within the reach of 90% of the population. On the other hand the government-run hospitals have a resource crunch with an overload of such patients. Standardized implants and surgeons experienced in spinal surgery are not universally available. Hence there is vast variation in the treatment offered to such patients. Ninety per cent of resources are utilized at places where only 10% spine injured patients are treated while 10% resources are available for 90% of the patients. We have no regionwise data which could be a guide to infrastructure development in a geographic area and thus no future projections are available. The treatment of a spinal trauma patient does not end with hospital management. Patients with neural compromise will continue to have problems of desensitized skin, bladder paralysis, autonomic disturbances and need to be cared for at home on a longterm basis. We need to rehabilitate them socially and give them vocational training so that they can earn their livelihood. This all looks a farfetched dream at present. But a beginning has to be made.

We can achieve improved outcome for spinal injury patients through better organization of spinal trauma care services. The guidelines have to be developed to set achievable standards for spinal trauma treatment services which could realistically be made available to almost every spinal trauma patient in this country. This includes human resources (staff and training) and physical resources (infrastructure, equipment and support). We need to collect epidemiological data to know the magnitude of the problem with average number of patients per lakh of the population, their demographic details, mode of trauma, mode of transportation, type of treatment given. The quantum of disability and handicap, cost of treatment in hospital and secondary cost incurred at home in followup will allow us to make provisions for future.

We need to have trained personnel to evaluate spinal trauma victims. The time elapsed between injury and safe transportation to the nearest spinal care unit should be minimum. Spinal trauma care requires an effective patient resuscitation, more sensitive diagnostic imaging, pharmacologic treatment to prevent secondary spinal cord injury, improved and timely preferred method of spinal column decompression and stabilization and better access to quality rehabilitation. There should be designated primary, secondary and tertiary spinal trauma centers for a given geographic area. The standardized protocol to clear the spine in a polytraumatized/ unconscious patient should be well documented and available at all causality services.

The objective of treatment in spinal trauma is to prevent secondary damage to the spinal cord, to achieve stability at fracture site, to decompress the spinal cord for possible neural recovery, to achieve shortest possible length of fusion and to rehabilitate the patients in their social milieu. Most of the literature used to frame guidelines for the management of spinal trauma comes from the west. This literature covers acute injury. It is unimaginable for them to see a patient of spinal trauma and spinal cord injury reaching late for treatment. The management strategies in late presentations, spinal trauma with complications and already operated cases requires an innovative approach. The treatment guidelines in such cases need to be made by a group of spine surgeons in this subcontinent only. The planning of surgical treatment in late presentation whether anterior surgery or posterior surgery alone or a combination of both, when combined which is first and the outcome in such cases for prognostication need to be described on the basis of analyzed data from our country and the subcontinent.

A scientific journal encompasses the responsibility of educating the orthopedic community about spinal trauma with the newer research. Whether to treat one such case by nonoperative method or by surgery and if surgery then whether along with stabilization should decompression of the spinal cord be performed or not; whether one should go for anterior or posterior or combined stabilization, should this be performed along with anterior column reconstruction by direct anterior bone graft or transpedicular bone graft or with bone graft substitute, whether the given patient will be able to tolerate operative morbidity and how to minimize the fused segment are various dilemmas in the mind of treating surgeons. We have attempted to answer some of the dilemmas. Wide variety of topics have been covered that include review articles on cervical spine trauma, thoracolumbar burst fracture and endoscopic surgery. It includes 16 original articles covering epidemiology, cervical spine trauma, classification of dorsolumbar trauma, indirect reduction of burst fracture, correction of kyphosis by short segment pedicle screw fixation with tanspedicular grafting by calcium phosphate, hydroxyapatite and transpedicular body augmenter.

I would like to thank the authors and reviewers for their timely submission of manuscripts, crisp and crystal clear reviews and prompt revisions. Dr. Ish Dhammi and Dr. Puneet Mishra, members of the editorial board have contributed immensely to various editorial steps to bring the symposium in this shape.

## References

[1] O'Connor RJ, Murrey PC (2006). Review of spinal cord injuries in Ireland. Spinal Cord.

[2] Botterell EH, Jousse AT, Kraus AS, Thompson MG, Wynne Jones M, Geviler WO (1975). A model for the future care of acute spinal cord injuries. Can J Neural Sci.

[3] Sekhon LH, Fehling MG (2001). Epidermology demographics and pathophysiology of acute spinal cord injury. Spine.

